# Supplementary material for: Application of Skyline for Analysis of Protein–Protein Interactions In Vivo
Source: Molecules. 2021 Nov 26;26(23):7170. doi: 10.3390/molecules26237170 (PMC8658920; doi:10.3390/molecules26237170)
Supplement: Supplementary file 1 [file molecules-26-07170-s001.zip › Supplementary data 1.pdf]

Supplementary data

Application of Skyline for analysis of protein-protein interactions *in vivo*

1.1. Building a Spectral Library in Skyline

Skyline is an open-source and freely available program, which can be downloaded from the next link https://skyline.ms/project/home/software/Skyline/begin.view

A new document was created in Skyline program, and peptide settings were adjusted as follows: Select **Settings > Peptide Settings**.

The settings appropriate to the experiment performed were selected by clicking the **Digestion** tab. Typical settings are:

- **Enzyme**: Trypsin [KR | P]
- **Max. missed cleavages**: 1,
- **Background proteome**: None

The modifications appropriate for the sample were selected by clicking on the **Modifications** tab. Modifications were added by clicking **Edit list > Add** and selecting modifications from the list in the **Edit Structural Modification** dialog. Spectral libraries were built based on database search results. The Mascot search engine was used to search in BAP1070 database, and the resulting DAT file was exported from the Mascot result page for processing in Skyline. To build a spectral library **Settings > Peptide Settings > Library > Build** was selected. An appropriate library name was typed into the Name field (BAPSox2_562). The preferred output path was specified by clicking the browse button next to the Output Path field. The **Keep redundant library** checkbox was selected. Skyline makes it possible for the user to inspect these spectra during a review of the results. The default Protein Prophet cut-offs core value was 0.95. **Next** and **Add Files** buttons was clicked. Then we went to the location where the database search result file, Mascot DAT file was saved, selected the file, and clicked **OK**. The file was added to the file list in the **Input Files** dialog.

To build the library, the checkbox(es) next to the search result file name(s) was(were) selected and then **Finish** button was clicked. The library was built in Skyline and a notification appeared briefly in the lower-left corner when the library has been successfully built. The library configuration was completed by selecting **Library** in the **Pick peptides matching** drop-down list on the **Peptide Settings > Library** tab. Two new files — BAPSx2_9hrbiotpulsonNidigest1_RA4_01_564.blib (containing the best-matching spectra) and BAPSx2_9hrbiotpulsonNidigest1_RA4_01_564.redundant.blib (containing all matching spectra) — should be located in the folder where Skyline saved the new spectral libraries.

The resulting library which contains the necessary retention time information was checked by clicking **View > Spectral Libraries**.

1.2. Configuring Transition Settings

The following Transition Settings were used for the analysis.

Prediction tab

- **Precursor mass > Monoisotopic**
- **Product ion mass > Monoisotopic**

Filter tab: **Precursor charges – 2, ion charges – 1, ion types – y,b. Product ion selection from ion 2 to 6 ions. Special ions – N-terminal to Proline**.

Library tab: **Ion match tolerance** was set to **0.5 Th**. Pick its most intense ions checkbox must be cleared.

Instrument tab:

Typical values are:

- **Min m/z > 50 Th**
- **Max m/z > 1500 Th**
- **Match tolerance m/z > 0.01 Th**
- **Firmware transition limit >** leave blank
- **Firmware inclusion limit >** leave blank

Full-Scan tab: MS1 filtering, Isotope peaks included – None; MS/MS filtering; Acquisition method: Targeted, Product mass analyzer: Centroided, Mass accuracy: 50 ppm.

1.3. Populating the Skyline Peptide Tree

. In order to add BAP1070 peptide for which quantitative data should be extracted a FASTA file (**File > Import > FASTA**) was imported. The import was started by double-clicking the FASTA file located in the corresponding folder. Alternatively, it can be done by copying complete FASTA sequences using a Notepad text-editor and then pasting them into Skyline.

1.4. Importing Raw Data into Skyline and Subsequent Filtering

In this step, raw data files were imported by selecting **File > Import > Results**.

In the **Import Results** dialog tab **Add single-injection replicates in file** to retain the default setting was selected. After selecting a raw data file(s) and clicking **Open**, Skyline started to import the file(s).

While the data is being imported, Skyline can be configured to optimize visualization of the filtered data (Figures 1S and 2S).

- Select **Settings > Integrate All**.
- Select **View > Peak Areas > Replicate Comparison** to open the Peak Areas pane.
- Right-click in the Peak Areas pane and select **Show Dot Product** and **Show Expected**.

1.5. Exporting results

In the **View** menu **Other grids > Result grids** were selected. All results, including total area analysis were exported as CSV file for subsequent calculation of biotinylation levels.

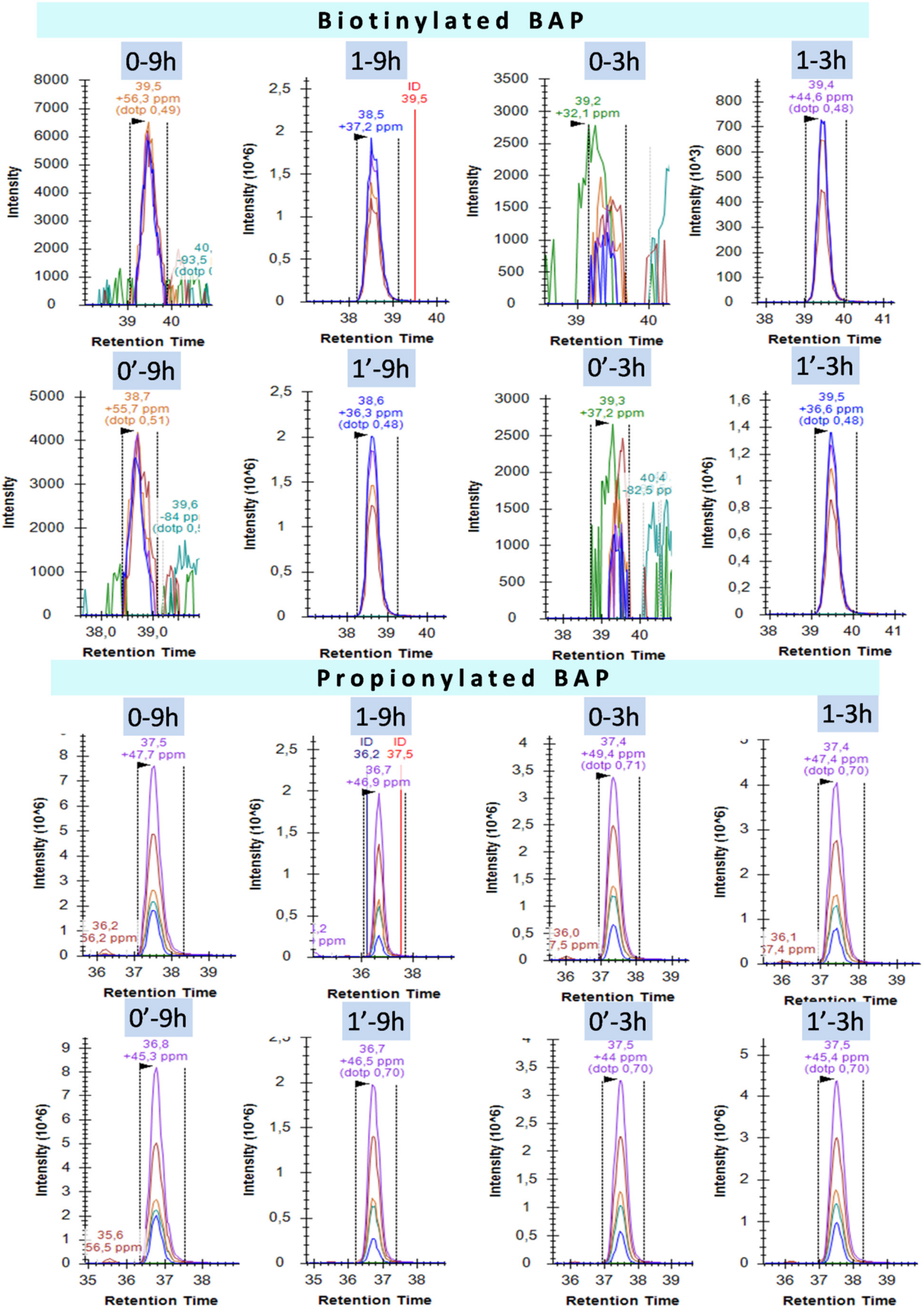

Figure S1. Extracted ion chromatograms (EICs) of the top four ranked y-ions of BAP. For clarity EICs only for two replicas are presented.

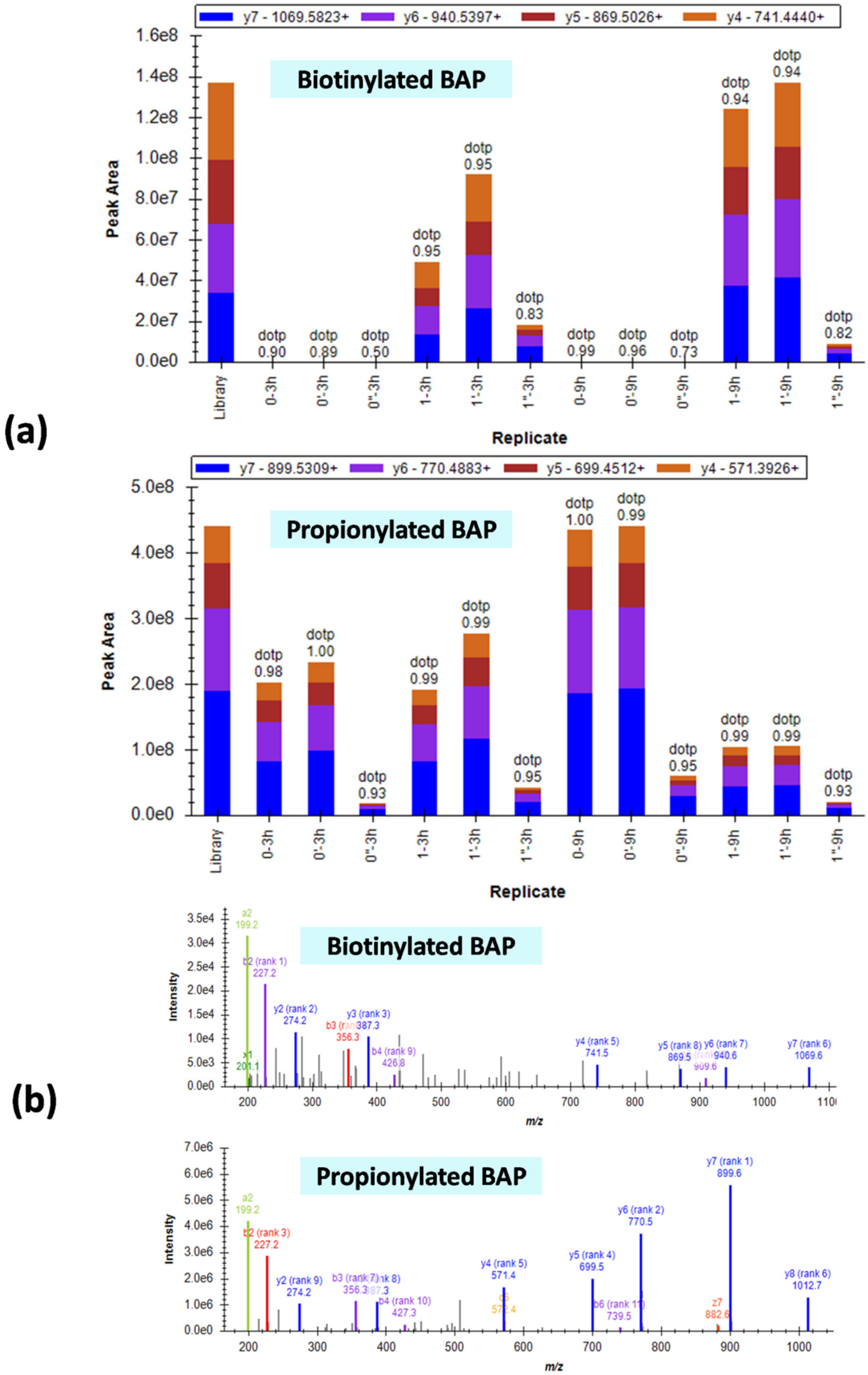

Figure S2. Relative quantification diagrams for biotinylated and propionylated forms of BAP after processing on Skyline; (a) Peak area graphs of experiments comparing the distribution of the 4 most intense y-type product ions in the MS/MS spectra of the BAP peptides. 0, 0′and 0′′ – three replicas of control experiment BAP-GFP+BirA-Oct4, 1, 1′ and 1′′ - three replicas of experiment with expression of interacting proteins BAP-Sox2+BirA-Oct4; (b) Skyline “library match” MS/MS spectra produced via CID fragmentation of the doubly charged precursor ion of the peptides ILEAQK(biotinyl)IVR (m/z 648.4190) and ILEAQK(propionyl)IVR (m/z 563.2001). 3h and 9h are biotinylation labeling times.
